# Supplementary material for: LucFlow: A method to measure Luciferase reporter expression in single cells
Source: PLoS One. 2023 Oct 4;18(10):e0292317. doi: 10.1371/journal.pone.0292317 (PMC10550117; doi:10.1371/journal.pone.0292317)
Supplement: Attachment — Submitted filename: Response to review.pdf [file pone.0292317.s014.pdf]

Responses to reviewer comments on manuscript #PONE-D-23-20181

Overview: We thank the editor and reviewer for their comments. We have revised the manuscript to address the comments and additional requirements of formatting style and funding disclosures. A point by point description of the response to reviewer's comments follows.

1. The reviewer asks us to justify the choice of Luciferase as a reporter.

   > It is not clear why to choose luciferase as a candidate protein. How about using GFP or other fluorescent proteins.

   While fluorescent proteins are certainly used as reporters, they are not suitable for all applications, especially investigations of dynamic gene regulation. Fluorescent proteins take minutes to hours for chromophore formation (Balleza et al., 2018) and have half lives on the order of hours to days (Snapp, 2009; Li et al., 1998) so that their fluorescence does not faithfully reproduce gene expression dynamics. Additionally, chromophore formation requires aerobic conditions, which may not be realizable in practice.

   Luciferase protein has been optimized for fast maturation and is suitable as a reporter of relatively rapid gene expression changes. Luciferase has other advantages. The protein is monomeric and does not require any post-translational modifications for activity. The assay has very high sensitivity and can be conducted with a simple and convenient protocol. Another reason to use Luciferase is that it is the most commonly used reporter; there are more than 40,000 articles matching the search term "Luciferase reporter assay" in PubMed. There are a large number of preexisting reporter vectors and cell lines and an improvement in the measurement methodology would be of immediate benefit to the field.

   Although the last paragraph of discussion had addressed some of these points, we now justify the use of Luciferase more explicitly in the introduction of the revised manuscript.

2. The reviewer suggests that we check whether Luciferase in dead cells retains activity.

   > It is suggested that authors can examine luciferase activity in dead cell transfected with luciferase proteins. Because luciferase activity assay is always used and quite sensitive in bulk cells.

   We thank the reviewer for this insightful comment. We did not detect any Luciferase protein by immunofluorescent staining (Fig. 2D,E) and concluded that dead cells do not have any functional Luciferase protein. The reviewer is wondering whether this undetectable protein might still retain enzymatic activity.

   Others have examined this question previously and found that increasing cell death results in lower Luciferase activity. For example, Mitsuki et al. (2016) show that increasing DMSO-induced cytotoxicity results in a proportional reduction of Luciferase activity. Similarly Didiot et al. (2011) documented a reduction in Luciferase activity as cell viability with increasing antibiotic concentration. Similar effects have been well documented by commercial vendors like Promega.

We have made this point clearer in the discussion of the revised manuscript.

3. There is no error bar in Figure 4.

Figure 4 plots the coefficient of variation (CV), which is the ratio of the standard deviation to the mean. The CV is already a measure of variance, and although one could compute the “variance of variance”, it would require a much bigger sample size for such an estimate to be reliable.

References

Enrique Balleza, J Mark Kim, and Philippe Cluzel. Systematic characterization of maturation time of fluorescent proteins in living cells. *Nat Methods*, 15(1):47–51, Jan 2018. doi: 10.1038/nmeth.4509.

Marie-Cecile Didiot, Sergio Serafini, Martin J Pfeifer, Frederick J King, and Christian N Parker. Multiplexed reporter gene assays: monitoring the cell viability and the compound kinetics on luciferase activity. *J Biomol Screen*, 16(7):786–93, Aug 2011. doi: 10.1177/1087057111407768.

X Li, X Zhao, Y Fang, X Jiang, T Duong, C Fan, C C Huang, and S R Kain. Generation of destabilized green fluorescent protein as a transcription reporter. *J Biol Chem*, 273(52):34970–5, Dec 1998. doi: 10.1074/jbc.273.52.34970.

Yu-Ya Mitsuki, Takuya Yamamoto, Fuminori Mizukoshi, Masatoshi Momota, Kazutaka Terahara, Kazuhisa Yoshimura, Shigeyoshi Harada, and Yasuko Tsunetsugu-Yokota. A novel dual luciferase assay for the simultaneous monitoring of hiv infection and cell viability. *J Virol Methods*, 231:25–33, May 2016. doi: 10.1016/j.jviromet.2016.02.006.

Erik Lee Snapp. Fluorescent proteins: a cell biologist’s user guide. *Trends Cell Biol*, 19(11):649–55, Nov 2009. doi: 10.1016/j.tcb.2009.08.002.
